# Top Questions in Uncomplicated, Non–*Staphylococcus aureus* Bacteremia

**DOI:** 10.1093/ofid/ofy087

**Published:** 2018-04-21

**Authors:** Jesse D Sutton, Sena Sayood, Emily S Spivak

**Affiliations:** 1 Department of Pharmacy, Veterans Affairs Salt Lake City Healthcare System, Salt Lake City, Utah; 2 Department of Medicine, University of Utah School of Medicine, Salt Lake City, Utah; 3 Department of Medicine, Division of Infectious Diseases, University of Utah School of Medicine & Veterans Affairs Salt Lake City Healthcare System, Salt Lake City, Utah

**Keywords:** bacteremia, oral antibiotics, duration of therapy, repeat blood cultures

## Abstract

The Infectious Diseases Society of America infection-specific guidelines provide limited guidance on the management of focal infections complicated by secondary bacteremias. We address the following 3 commonly encountered questions and management considerations regarding uncomplicated bacteremia not due to *Staphylococcus aureus*: the role and choice of oral antibiotics focusing on oral beta-lactams, the shortest effective duration of therapy, and the role of repeat blood cultures.

Bacteremia complicates approximately 2%–25% of focal infections, such as pneumonia and urinary tract infection (UTI), managed in the hospital and may be associated with poor outcomes [1, 2]. Most infection-specific Infectious Diseases Society of America (IDSA) guidelines do not provide direct management recommendations for choice or route of antibiotic administration, duration of therapy, or use of repeat blood cultures in secondary bacteremias [3, 4]. Until recently, few studies evaluated these common management considerations, and the available literature suggests that considerable practice variation exists [5–7]. Use of the least invasive route of antibiotic administration and the shortest effective duration of therapy are imperative given the relationship between route and duration of antibiotic exposure and adverse drug events, *Clostridium difficile* infections, and antibiotic resistance [8–11]. Given these considerations, we aim to briefly summarize the existing literature regarding 3 management considerations in uncomplicated bacteremia not due to *Staphylococcus aureus* in adults: (1) the role and choice of oral antibiotics focusing on beta-lactams, (2) the shortest effective duration of therapy, and (3) the role of repeat blood cultures. No standard definition of uncomplicated bacteremia exists; therefore, we use uncomplicated bacteremia to refer to immunocompetent, bacteremic patients without an uncontrolled source of infection or deep-seated infection for which treatment durations greater than 2 weeks are routinely recommended [3, 12, 13].

## WHAT IS THE ROLE OF ORAL ANTIBIOTICS?

Randomized trials, observational studies, and pharmacokinetic-pharmacodynamic principles support the efficacy of high-bioavailability oral agents or agents that achieve approximately equivalent serum concentrations when administered orally or intravenously (eg, fluoroquinolones, oxazolidinones, trimethoprim-sulfamethoxazole) for the treatment of invasive infections including bacteremia from a variety of organisms such as *Enterococcus* species*, Streptococcus* spp., Enterobacteriaceae, and *Pseudomonas aeruginosa* [14–23]. However, antibiotic resistance and the risk of adverse effects often limit the use of these agents, highlighting the need for additional treatment options [24–28]. Oral beta-lactams are well tolerated and retain activity against several relevant organisms that cause bacteremia, such as Enterobacteriaceae and streptococci [25, 28]. However, there are concerns regarding the efficacy of oral beta-lactams for bacteremia because they result in substantially lower serum concentrations compared with intravenous beta-lactams [29, 30]. Despite lower serum concentrations, oral beta-lactams may be effective in specific scenarios given the multifactorial nature and interpatient variability in achieving therapeutic drug concentrations. Unfortunately, there is a lack of robust microbiologic and pharmacokinetic information in varying patient populations to guide the use of oral beta-lactams in many scenarios involving bacteremia.

The IDSA’s community-acquired pneumonia (CAP) guidelines are the only infection-specific guidelines to address the use of oral antibiotics in the setting of bacteremia [4]. The IDSA CAP guidelines suggest that intravenous to oral conversion is safe and effective in the setting of *Streptococcus pneumoniae* bacteremia [4]. The efficacy of both oral beta-lactams and high-bioavailability oral agents in *S. pneumoniae* bacteremia from CAP is supported by observational studies and subsets of randomized trials [31–37]. Major areas of uncertainty relate to the use of oral beta-lactams for focal infections with bacteremia caused by Enterobacteriaceae*, Enterococcus* spp., or *Streptococcus* spp. apart from pneumonia. Due to an absence of clinical outcomes data regarding use of oral beta-lactams for bacteremia caused by *Enterococcus* spp. or *Streptococcus* spp., we will focus on the use of oral beta-lactams for Enterobacteriaceae bacteremia.

There are no randomized controlled trials (RCTs) directly addressing the role of oral beta-lactams in the treatment of Enterobacteriaceae bacteremia; however, some information can be gleaned from RCTs reporting outcomes in the subset of bacteremic patients. First, limited RCT data suggest that use of oral beta-lactams alone without initial intravenous antibiotics is associated with higher clinical and microbiologic recurrences in the setting of complicated UTI and pyelonephritis with or without Enterobacteriaceae bacteremia [38, 39]. Second, clinical cure rates were either consistently greater than 90% or did not differ between bacteremic and nonbacteremic patients in several RCTs involving definitive oral beta-lactam treatment after initial intravenous therapy [40–46]. This observation is based on outcomes directly reported in approximately 50 patients, as well as generic statements in 2 RCTs for which outcomes were not specifically stated [40–46].

Three retrospective cohort studies have more directly investigated the role of oral therapy including beta-lactams in the setting of primarily Enterobacteriaceae bacteremia, with somewhat conflicting results [47–49]. However, reported success rates exceeded 85% in all 3 studies. All 3 studies included a group of patients who received definitive oral therapy after initial intravenous antibiotics for a median of 3–5 days. The most common source of secondary bacteremia was UTI (≥70%), followed by intra-abdominal or biliary infection [47–49]. Mercuro and colleagues performed a single-center study comparing definitive therapy with oral beta-lactams (n = 84) with fluoroquinolones (n = 140) [47]. Clinical success rates were equivalent when comparing oral beta-lactams (86.9%) with fluoroquinolones (87.1%; odds ratio [OR], 1.24; 95% confidence interval [CI], 0.57–2.71) and when intravenous to oral switch occurred within the first 3 days vs later [47]. Kutob and colleagues compared definitive therapy with antibiotics categorized as low (ie, oral beta-lactams, n = 77), moderate (ie, ciprofloxacin, trimethoprim-sulfamethoxazole, n = 179), or high bioavailability (ie, levofloxacin, n = 106) [48]. Failure occurred in 14% of the low, 12% of the moderate, and 2% of the high bioavailability. Both low- (hazard ratio [HR], 7.7; 95% CI, 1.9–51.5) and moderate- (HR, 5.9; 95% CI, 1.6–38.5) compared with high-bioavailability agents were associated with increased risk of failure, and failure occurred earlier in the low bioavailability group [48]. Lastly, Rieger and colleagues performed a study comparing the efficacy of intravenous only (n = 106) vs intravenous to oral treatment (n = 135) for bacteremic UTIs [49]. Treatment failure occurred in 3.8% of the intravenous only vs 8.2% of the intravenous to oral group (*P* = .19). No specific information was provided regarding outcomes in the 19% of patients receiving oral beta-lactams [49].

There are clinical and pharmacokinetic-pharmacodynamic data supporting the safety and efficacy of high-bioavailability agents (eg, fluoroquinolones, oxazolidinones, trimethoprim-sulfamethoxazole) for the treatment of uncomplicated bacteremia when confirmed by susceptibility testing and in the absence of factors diminishing oral absorption. Additionally, oral beta-lactams can be used for uncomplicated *S. pneumoniae* bacteremia especially due to pneumonia. Further clinical and pharmacokinetic data are needed to guide the optimal use of oral beta-lactams for uncomplicated Enterobacteriaceae bacteremia; however, available RCT and observational data suggest that conversion from initial intravenous to definitive oral beta-lactam therapy results in high success rates in the appropriately selected patient. It is unclear if high success rates result from the efficacy of oral beta-lactams or the initial course of intravenous antibiotics, as the shortest effective duration of therapy is unknown. Based on the available data, it is reasonable to consider oral beta-lactams as definitive therapy for uncomplicated Enterobacteriaceae bacteremia in patients who have responded clinically to intravenous therapy, particularly in the setting of a pathogen with sufficiently low minimum inhibitory concentration and a patient who is not predisposed to low beta-lactam concentrations (eg, rapid drug elimination, increased volume of distribution). There is a lack of clinical data to guide the use of oral beta-lactams for bacteremia secondary to *Streptococcus* spp., *Enterococcus* spp., and alternate infection sources.

## WHAT IS THE SHORTEST EFFECTIVE DURATION OF THERAPY?

There is growing evidence that short (≤7 days) as compared with longer treatment durations are equally effective for uncomplicated infections and associated with fewer negative consequences of antibiotic use [8, 10, 50–52]. It is unclear if this evidence applies to patients with secondary bacteremia. The intravascular catheter-related infection guidelines are the IDSA’s only infection-specific guideline to provide a recommendation for duration of therapy in the setting of bacteremia [3]. The recommended duration for uncomplicated Gram-negative bacilli and *Enterococcus* spp. ranges from 7 to 14 days [3]. Recommendations from non-IDSA guidelines range from 7 to at least 14 days depending on the organism and source [53, 54]. In the absence of clear recommendations, the most commonly used duration is 14 days [5–7, 12].

There are no RCTs published to date comparing durations specifically in bacteremic patients. Limited data exist from RCTs comparing different durations for focal infections and reporting outcomes in the subset of bacteremic patients [55]. A meta-analysis of RCTs comparing the same antibiotic for 5–7 days vs a longer duration identified only 7 trials reporting an outcome in 155 bacteremic patients. The sources of infection were neonatal bacteremia (43%), pneumonia (26%), spontaneous bacterial peritonitis (26%), and pyelonephritis (6%). There were no differences in the rates of clinical cure (risk ratio [RR], 0.88; 95% CI, 0.77–1.01), microbiologic cure (RR, 1.05; 95% CI, 0.91–1.21), or survival (RR, 0.97; 95% CI, 0.76–1.23) [55]. We reviewed additional systematic reviews and meta-analyses of RCTs that included the most common focal infections in hospitalized patients, compared a short (≤7 days) and long (>7 days) duration of therapy, and reported outcomes in the subset of bacteremic patients [55–62]. Six complicated UTI or pyelonephritis RCTs included approximately 140 bacteremic patients [16, 46, 63–66]. A fluoroquinolone was used for a short duration in 5 RCTs, and intravenous to oral beta-lactam was used in 1 RCT. There were no reported differences in clinical cure rates between patients treated for a short or long duration or between bacteremic and nonbacteremic patients [16, 46, 57, 63–66]. Six nonazithromycin CAP RCTs included approximately 90 patients with *S. pneumoniae* bacteremia, with no differences in clinical efficacy in patients treated with a short vs long duration or in bacteremic vs nonbacteremic patients [31, 35, 67–70]. A short course treatment consisted of a beta-lactam in 3 trials, fluoroquinolone in 2 trials, and ketolide in 1 trial [31, 35, 67–70]. Consistent with the previously cited meta-analysis, there are few bacteremic patients with outcomes available to compare short vs longer duration; however, available RCTs suggest that a shorter duration is as effective as longer durations in Enterobacteriaceae bacteremia secondary to UTIs and *S. pneumoniae* bacteremia secondary to CAP.

Four published retrospective studies compared a short vs long duration for secondary bacteremias [12, 13, 71, 72]. Chotiprasitsakul and colleagues performed a multicenter, propensity score–matched cohort study comparing 30-day mortality in patients with Enterobacteriaceae bacteremia who received antibiotics for 6 to 10 days (n = 385) vs 11 to 16 days (n = 385) [71]. Median durations were (interquartile range [IQR]) 8 (7–9) and 15 (13–15) days. UTI was the most common source (37%). Short course was not associated with increased 30-day mortality (9.6% vs 10.1%; HR, 1.00; 95% CI, 0.62–1.63) or 30-day recurrent bloodstream infections (1.3% vs 2.3%; OR, 1.32; 95% CI, 0.48–3.41) [71]. Nelson and colleagues performed a multicenter cohort study comparing clinical failure in patients with Gram-negative bacteremia receiving antibiotics for 7–10 days (n = 117) vs longer (n = 294) [13]. Median durations (IQR) were 9 (7–10) and 13 (12–15) days. Urine was the most common source (69%), and Enterobacteriaceae was the most common pathogen (90%). Treatment failure was associated with shorter course therapy (HR, 2.60; 95% CI, 1.20–5.53), with the difference driven by 90-day mortality (8.2% vs 3.3%, *P* = .04), not 90-day recurrent infection (6.7% vs 6.5%, *P* = .93). Median time to treatment failure (IQR) was 36 (10–69) days [13]. Daneman and colleagues performed a multicenter, propensity score–matched cohort study in critically ill patients with uncomplicated bacteremia from a wide distribution of sources and pathogens. Two hundred twenty-two matched pairs were included. The median durations (IQR) were 7 (4–8) and 15 days (14–20). Mortality (27% vs 29%; RR, 0.94; 95% CI, 0.70–1.26) and recurrent bacteremia (6% vs 8%, *P* = .29) were not different in patients receiving short and long durations, respectively [12]. Lastly, Doi and colleagues performed a retrospective single-center study comparing 30-day mortality in patients with bacteremia secondary to cholangitis who received antibiotics for 7 or fewer days (n = 86) compared with longer (n = 177) [72]. All patients had source control, and median durations of therapy were 6 and 12 days. The most common organisms were Gram-negatives (87% vs 89%), but 13% and 27% had polymicrobial bacteremia in the short and long groups, respectively. The 30-day mortality rates were 5% and 6% (OR, 0.82; 95% CI, 0.18–2.95) [72]. In summary, the 3 studies assessing mortality within 30 days or less reported no difference between the different treatment durations [12, 71, 72], while the lone study assessing outcomes within 90 days did identify a difference [13]. While the relative merits of each outcome time frame have been debated, using a 90-day end point increases the likelihood of capturing mortality related to underlying comorbidities rather than the duration of antibiotic treatment for an acute bacteremia [73, 74]. Additional more limited evidence suggests that shorter durations may be as effective as longer durations for a variety of bacteremic sources [47, 75–80].

In summary, the optimal duration of therapy for uncomplicated bacteremia is understudied. More data are needed as a basis for the shortest effective duration. There are multiple ongoing RCTs comparing 7 vs 14 days in patients with bacteremia from various sources and organisms [81–85]. Until results are available, available clinical trial and observational literature suggest that shorter treatment durations are as safe and effective as longer durations for uncomplicated Enterobacteriaceae bacteremia and *S. pneumoniae* bacteremia from pneumonia. There is a lack of comparative data investigating the optimal treatment duration for non-Enterobacteriaceae Gram-negative organisms (eg, *P. aeruginosa, Acinetobacter* spp.), *Enterococcus* spp., and Streptococcus spp., as these organisms were not present or were present in relatively lower numbers in the previously discussed studies. Of note, there are limited published data to support the common practice of treating for 14 days for most clinical scenarios of uncomplicated bacteremia not due to *S. aureus*.

## WHAT IS THE ROLE OF REPEAT BLOOD CULTURES?

With the exception of *S. aureus* bacteremia, the utility of repeat blood cultures is not well defined. Studies examining this question are small, single-center retrospective studies [86–88]. However, the small amount of existing data suggest limited utility in repeat blood cultures in cases of Gram-negative bacteremia or bacteremia secondary to UTIs and skin and soft tissue infections (SSTIs) [86–88]. The largest study to date investigating the utility of repeat cultures in bacteremia included 701 repeat blood cultures, with persistent bacteremia reported in 17% [86]. Persistent bacteremia was defined as repeat blood culture positivity with the same organism 2–7 days following the initial culture. Of the persistent bacteremias, 76% were Gram-positive organisms, and 54% were from endovascular or bone and joint sources. A nested case-control study was performed comparing patients with persistent (n = 118) vs cleared bacteremia (n = 118). Gram-positive organism, endovascular source, and epidural source were associated with persistent bacteremia in multivariate analysis. Genitourinary source, *Escherichia coli,* and streptococci were associated with a lower risk [86]. An additional retrospective study performed by Canzoneri and colleagues included 383 repeat blood cultures with an overall positive yield of 14% [87]. Seventy-eight percent of repeat positives were Gram-positive cocci, while 15% were Gram-negatives alone. There was a negative association with persistent bacteremia and UTI or SSTI source. Five follow-up blood cultures were needed to detect 1 positive, but this number increased to 17 when looking only at the Gram-negative cases [87]. A smaller study with 38 repeat blood cultures from bacteremic UTIs showed a repeat positive yield of only 8%, and all repeat positives were secondary to Gram-positive organisms [88]. Of note, prescribed antibiotic durations were significantly longer in patients with repeat blood cultures performed (15 vs 12 days) [88]. The necessity of repeat blood cultures is further called in to question when examining evidence showing the limited clinical utility of *initial* blood cultures in the cases of *S. pneumoniae* pneumonia and pyelonephritis [89–91]. In these retrospective studies, bacteremia was not correlated with increased mortality or morbidity [89–91]. Additionally, the results of initial positive blood cultures had no effect on treatment choice in the case of UTI [91].

Given the low yield of repeat blood cultures in uncomplicated non–*S. aureus* bacteremia, the unclear impact on clinical decision-making, and the potential correlation with increased antibiotic days, there appears to be limited added value in the clinical practice of routinely obtaining repeat blood cultures for the purpose of documenting bloodstream clearance. Therefore, we suggest considering the source of infection when deciding whether to repeat blood cultures because the available literature suggests that repeat blood cultures are low yield in the setting of bacteremia secondary to UTIs and SSTIs. Similarly, we would discourage routine documentation of blood culture clearance with Gram-negative bacteremia. We suggest obtaining repeat blood cultures when the source of bacteremia is unknown or there is a lack of clinical improvement, raising concern for complicated infection. Further studies focusing on specific organisms and sources of infectious would be beneficial given the low number of organisms such as *P. aeruginosa* in these studies.

## SUMMARY

There is currently a lack of extensive evidence to establish strong recommendations for common management considerations in uncomplicated bacteremia. However, it is imperative that new management approaches be considered that balance optimizing clinical outcomes and limiting unintended consequences of excessive antibiotic use. To that end, the available evidence suggests that short treatment durations are safe and effective in the setting of uncomplicated Enterobacteriaceae and *S. pneumoniae* bacteremia, and there appears to be limited utility for routine repeat blood cultures to document bloodstream clearance in the setting of clinical improvement and minimal concern for complicated infection. High-bioavailability oral agents can be reliably used for uncomplicated bacteremia, and oral beta-lactams can be considered after initial intravenous treatment for select patients with uncomplicated Enterobacteriaceae or *S. pneumoniae* bacteremia.
